# Organoids technology in cancer research: from basic applications to advanced *ex vivo* models

**DOI:** 10.3389/fcell.2025.1569337

**Published:** 2025-05-22

**Authors:** Luca Varinelli, Oscar Illescas, Ewelina Julia Lorenc, Davide Battistessa, Marzia Di Bella, Susanna Zanutto, Manuela Gariboldi

**Affiliations:** Molecular Epigenomics Unit, Department of Experimental Oncology, Fondazione IRCCS Istituto Nazionale Tumori, Milan, Italy

**Keywords:** cancer organoids, personalized therapy, extracellular matrix (ECM), decellularized matrix, *ex vivo* cancer models, drug screening

## Abstract

Patient-derived organoids (PDOs) are tridimensional cultures derived from the stem component of a tissue. They preserve the genetic and phenotypic characteristics of the tissue of origin, and represent valuable *in vitro* models for drug screening, biomarker discovery, cell therapy and genetic modification. Importantly, PDOs reproduce the tumor behavior and can predict therapeutic responses, making them relevant for clinical applications for personalized therapies. PDOs may also be used for studying the interactions between cancer cells and the tumor microenvironment (TME). These interactions are driven by biochemical factors released by the cells, and biomechanical events such as the remodeling of the extracellular matrix (ECM). In recent years, it has become evident that the interactions between cancer cells and the TME have an impact on tumor development and on the efficacy of cancer therapy Therefore, targeting both tumor cells and the TME may improve patient response to treatment. Most PDO culture protocols are limited to epithelial cells. However, recent advances such as use of decellularized ECM (dECM) scaffolds have allowed for the development of *in vivo*-like environments that host diverse cell types, both normal and pathological, in a tridimensional (3D) manner that closely mimics the complexity of the TME. dECM-based models effectively replicate the interactions between tumor cells, ECM and the microenvironment, are easy to analyze and adaptable for drug testing. By incorporating TME components and therapeutic agents, these models offer an advanced platform for preclinical testing.

## 1 Introduction

A major challenge in developing new therapies is translating scientific knowledge from the laboratory to the clinical practice (Drost and Clevers, 2018). Many current cancer models are unable to fully recapitulate patient tumors, leading to the development of therapies that work in preclinical models but fail in patients (Liu et al., 2023; Kamb, 2005). Common models include patient-derived cancer cell lines and patient-derived tumor xenografts (PDTX). Two-dimensional (2D) cancer cell lines, typically developed from patient tumors, have several drawbacks. They are inefficient to generate, and often lose the tumor’s genetic diversity (Habanjar et al., 2021). Most importantly, 2D lines lack the tumor’s stromal compartment (Cheon and Orsulic, 2011). PDTXs, which are grown by transplanting patient tumor tissue into immunodeficient mice, better mimic human tumor biology (Drost and Clevers, 2018). However, they are expensive, time-consuming, and face challenges such as engraftment inefficiency for certain tumor types (Kim et al., 2020). Moreover, PDTXs may evolve differently in mice than in humans, limiting their relevance (Ben-David et al., 2017).

In response to these limitations, 3D culture methods have been developed to create more accurate models of human tissue. These models, known as patient-derived organoids (PDOs), can self-organize into complex structures that better represent both healthy and cancerous tissues (Fatehullah et al., 2016; Jensen and Teng, 2020; Paradiso et al., 2021).

## 2 Patient-derived 3D organoids cultures

**FIGURE 1:**
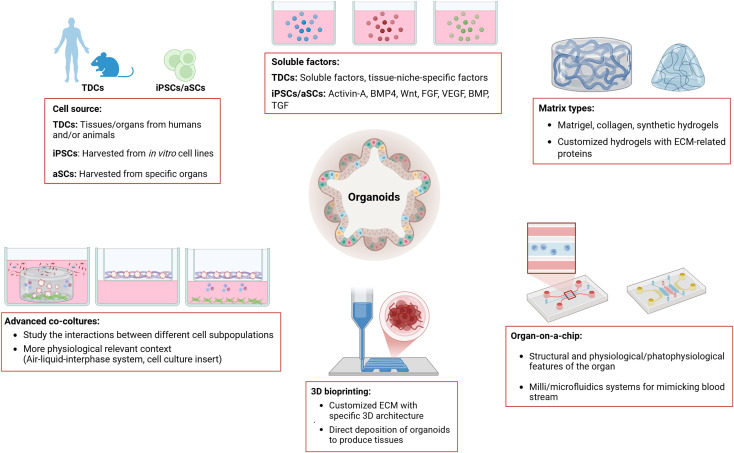
This figure summarizes the main methods used for organoid culture. Organoids can be developed from tissue-derived cells (TDCs), from adult induced-pluripotent stem cells (iPSCs) or from organ-restricted adult stem cells (aSCs). Depending on the source of the cells used to generate the organoids, the growth and expansion media need to be supplemented with growth factors and/or specific soluble factors. Organoids are grown in matrices of either animal or synthetic origin, which provide structural support and promote cell aggregation in a 3D manner. Organoids can be developed, grown and expanded using advanced culture techniques, such as Organ-on-a-chip technology, 3D bioprinting, and various co-culture methods (i.e., Air-liquid-interface system, cell-culture insert). Key features of the different organoid culture methods are highlighted in the figure (see boxes). Legend: TDCs, Tissue-derived Cells; iPSCs, induced-Pluripotent Stem Cells; BMP4, Bone Morphogenetic Protein 4; Wnt, Wnt protein; FGF, Fibroblast Growth Factor; VEGF, Vascular Endothelial Growth Factor; BMP, Bone Morphogenic Protein; TGF, Transforming Growth Factor; ECM, Extracellular Matrix.

PDOs have been widely used in developmental biology studies from the 1960s and 1980s to investigate organogenesis through cell dissociation and reaggregation experiments (Lancaster and Knoblich, 2014; Kim et al., 2022). Over the past decade, PDOs have gained significant attention in the scientific community because of their unique properties. PDOs are organ-like structures composed by different cell subtypes organized through spatially restricted lineage commitment (Eiraku and Sasai, 2012). PDOs can be derived from embryonic pluripotent stem (ES) cells, organ-restricted adult stem cells (aSCs), or synthetic induced pluripotent stem cells (iPSCs), (Figure 1). Particularly, PDOs developed from iPSCs are able to capture the theoretically limitless expansion potential of stem cells *in vitro* (Tang et al., 2022). Instead, aSCs were thought to be unable to proliferate outside the organism, until the development of growth factor cocktails that mimic the stem cell niches of different organs, which has allowed their expansion *in vitro* (Clevers, 2016). Still, both iPSCs and aSCs, when induced to differentiate *in vitro*, are able to self-organize into structures that mirror key aspects of the tissue from which they are derived (Figure 1), (Clevers, 2016; Fatehullah et al., 2016; Jensen and Teng, 2020).

### 2.1 iPSC-derived organoids

**FIGURE 2:**
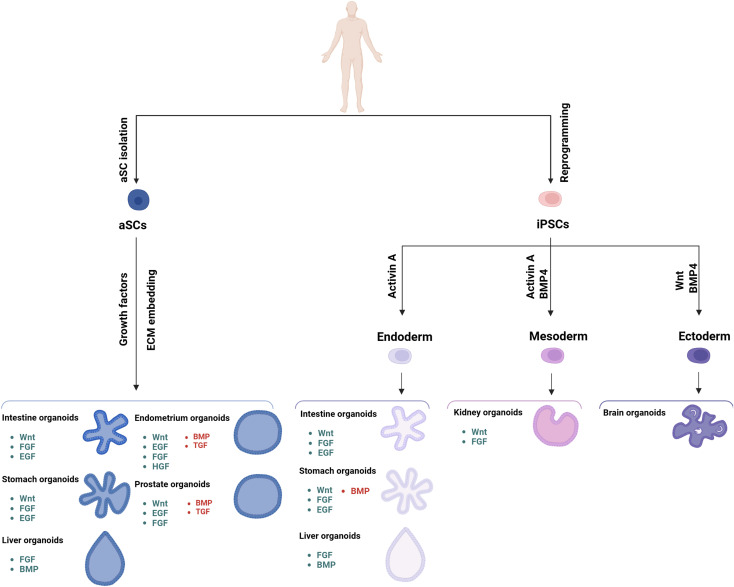
Schematic representation of the different organoids that can be developed from iPSC and aSC cells, along with the various growth factors required for their development. The signaling components essential for guided differentiation and niche function are shown, with activated signaling pathways shown in green, and inhibited ones in red. Key factors include BMP, bone morphogenetic protein; EGF, epidermal growth factor; FGF, fibroblast growth factors; HGF, hepatocyte growth factor; IGF, insulin-like growth factor; ROCK, RHO-associated protein kinase; TGF, transforming growth factor; VEGF, vascular endothelial growth factor.

ES and iPSCs organoids have been generated from various organs, including brain, retina, pancreas, stomach, lung, thyroid, liver, and intestine (Lancaster et al., 2013; Norrie et al., 2021; Cox et al., 2019; Yan et al., 2018; Sachs et al., 2019; Ogundipe et al., 2021; Ramli et al., 2020; van de Wetering et al., 2015). In the intestine, signals mediated by the Wnt (WNT) and the fibroblast growth factor (FGF) protein families can induce posterior endoderm patterning, hindgut and intestinal morphogenesis, differentiation, and growth. Specifically, the combined activity of the Wnt protein 3A (WNT3A) and fibroblast growth factor 4 (FGF4) is required for hindgut differentiation, while FGF4 alone is sufficient to promote hindgut morphogenesis (Figures 1, 2). For these reasons, supplementation of culture media with these factors is essential for developing organoids from the midgut and small intestine (Spence et al., 2011). Human-derived mid and hindgut iPSCs require culture media supplemented with FGF4, WNT3A, and activin, which promotes ES cells differentiation, to maintain their self-renewal ability and support long-term culture without feeder cells (Figures 1, 2).

To reproduce the mid/hindgut structure, organoids require both the factors that mimic the intestinal niche and a scaffold, such as Matrigel (Corning). Matrigel is a solubilized basement membrane preparation derived from mice, rich in extracellular matrix proteins, which enables cells to grow in three dimensions (Figure 1). (Sato et al., 2011). These organoids are cultured for 1–3 months to obtain villus and crypt-like structures with proliferative zones containing all major epithelial cell subtypes, and including polarized epithelial patterns, areas rich in myofibroblasts and smooth muscle cells surrounding epithelial cells (Figure 2), (McCracken et al., 2014). Following the development of iPSC-derived intestinal organoids, several protocols have been established to obtain them from other human tissues (Koo and Clevers, 2014). Each protocol has been developed with a deep biological understanding of the organ type, guided by extensive pilot studies to optimize efficacy (Le Savage et al., 2022; Varinelli et al., 2024a).

### 2.2 aSC-derived organoids

The first protocol to obtain organoids from aSCs was developed using intestinal tissue, as the molecular mechanisms regulating intestinal stem cell turnover are well understood. WNT has emerged as a key driver of epithelial aSC growth (Clevers, 2016), inducing the secretion of R-spondin-1 protein (RSPO1), the ligand of the leucine-rich repeat-containing G protein-coupled receptor 5 (LGR5) which is expressed in most aSCs (Koo and Clevers, 2014). WNT proteins is a large family of secreted glycoproteins with nineteen different proteins in humans, suggesting a daunting complexity of signaling regulation (Figures 1, 2). Indeed, the WNT pathway regulates critical processes such as cell fate determination, migration and polarity, neural patterning, and organogenesis during embryonic development (Komiya and Habas, 2008). Wnt activators like WNT3A, RSPO1, or small molecules such as glycogen synthase kinase three protein (GSK3) inhibitors, are key components of many aSC-derived organoid culture protocols (Figure 2), (Corsini and Knoblich, 2022). These studies have enabled the generation of PDOs from the pancreas, prostate, esophagus, ovary, liver, kidney and breast (Broutier et al., 2016; Boj et al., 2015; Drost et al., 2016; Kopper et al., 2019; Homan et al., 2019; Sachs et al., 2018).

The need of these factors to mimic the niche environment can be explained by the biological characteristics of the intestinal epithelium, which is constantly under stress due to food digestion and nutrient metabolism, and undergoes rapid and high turnover. This is driven by highly proliferating stem cells at the base of the intestinal villi, known as transiently amplified (TA) cells, which form the stem cell crypt. A key feature of TA cells is the expression of LGR5, a specific marker of intestinal stem cells (Barker et al., 2010). The stem cells occupy the apical portions of the crypt, move to the villus sides after differentiation, induced by bone morphogenetic protein 4 (BMP4) signaling, and ascend to the luminal end, where they eventually undergo cell death and are replaced by new differentiated cells within 5 days (Clevers, 2016). Several types of differentiated epithelial cells are found in the villi surface, including enterocytes for nutrients absorption, secretory cells like Paneth, goblet, enteroendocrine, tuft cells, and microfold cells forming Peyer’s plaques (Clevers, 2013).

Stem cell differentiation and fate in the intestinal crypt are mainly regulated by four signaling cascades: the neurogenic locus notch (NOTCH), epidermal growth factor-mediated (EGF), bone morphogenesis protein (BMP) and WNT pathways. WNT signaling drives the proliferation of both non-actively proliferating stem cells and TA cells, while NOTCH maintains these cells in an undifferentiated state; blocking NOTCH protein leads to differentiation into goblet cells. EGF-mediated signaling strongly stimulates proliferation of both stem and TA cells, while BMPs, which are active along the apical regions of the intestinal villi, must be inhibited to create a crypt-permissive environment (Clevers, 2013), (Figure 2).

LGR5-positive crypt stem cells can undergo hundreds of cell divisions *in vivo*. Current protocols for culturing intestinal organoids rely on the mechanical and enzymatic isolation of crypts, or single LGR5-positive cells, which are then cultured in Matrigel to provide structural support (Sato et al., 2009). These cells are cultured in serum-free media supplemented with recombinant proteins RSPO1, EGF, Noggin, and WNT3A, which is necessary to obtain PDOs from the non-tumor epithelium (Clevers, 2016). Finally, to obtain long-term colon PDO cultures, inhibitors of the ALK tyrosine kinase receptor (ALK) and the ribonuclease P subunit 38 proteins (p38) are added to block BMP signaling (Clevers, 2013; Clevers, 2016) (Figures 1, 2). The resulting PDOs are heterogeneous, displaying all major subtypes of epithelial cells found *in vivo* (Sato et al., 2009). These organoids feature a highly polarized epithelium with a central lumen and crypt-like structures protruding outwards, with the basal surface facing the Matrigel, and enterocytes forming the luminal surface. Secretory cells are located inside the lumen (Sato et al., 2009; Sato et al., 2011).

## 3 Role of organoids in translational cancer research

### 3.1 Development of cancer PDO biobanks

Successful strategies have been developed to obtain PDOs from a wide variety of tumors (Clevers, 2016), and these organoids have been shown to accurately recapitulate key phenotypic and genetic features of the tumor of origin (Drost and Clevers, 2018). PDOs are derived from a pool of tumor cells grown under specific selective conditions that mirror the characteristics of the original tumor. For example, PDOs from tumors with activating mutations in the WNT pathway, common in colorectal cancer (CRC)^1^ can be generated without supplementation of WNT and RSPO1-related factors (Sato et al., 2011). Conversely, tumors with mutations in the EGF receptor (EGFR) are grown without EGF supplementation (Fujii et al., 2016).

**FIGURE 3:**
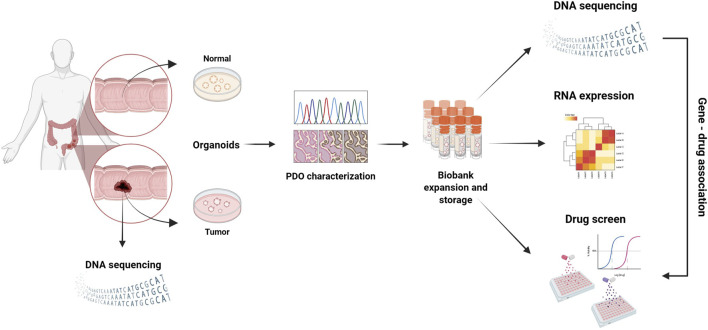
3D PDO cultures derived from various biobanks, which mimic the key features of different cancer types, are used for drug screening to identify potential therapeutic markers that could help personalize patient treatment.

Large collections of PDOs from cancer tissues, along with matched healthy controls, have led to the creation of PDO biobanks, which are being tested to predict personalized responses to specific drug treatments (Figure 3). For example, studies of PDOs derived from rectal cancer from different biobanks, have shown a correlation between the PDO response to standard drug treatments and the clinical responses of patients from whom they were derived (Yao et al., 2020; Luo et al., 2023; van de Wetering et al., 2015; Mo et al., 2022; Farin et al., 2023). PDOs can also be xenotransplanted into immunocompromised mice, where they maintain stable mutational profile and histopathological characteristics (Fujii et al., 2016), and biobanks of PDO xenotransplants have been used to validate pharmacological responses in a more complex *in vivo* context (Dekkers et al., 2021; Lago et al., 2023; Xu et al., 2022; Dreyer et al., 2021; Beshiri et al., 2018) (Figure 3). CRC-PDO biobanks have also demonstrated the susceptibility of certain CRC subtypes to inhibitors targeting non-canonical *Wnt* pathways (Chen et al., 2009), and have shown that drug responses can be independent of the tumor’s genetic landscape (Maimets et al., 2016). Furthermore, PDOs can be used to identify the biological mechanisms underlying the disease (Figure 3). For example, a study on a collection of 55 CRC-PDOs revealed that the growth of specific tumor subtypes depends on different signaling pathways (Fujii et al., 2016). Also, a drug screening targeting receptor tyrosine-protein kinase erbB2 (HER2) signaling, in a biobank representing breast cancer heterogeneity with over 100 PDOs, demonstrated that sensitivity to drug treatment correlates with HER2 status (Sachs et al., 2018).

A major challenge now is expanding PDO biobanks and linking them, to enhance statistical representativeness and correlate genetic markers with drug sensitivity. The Human Cancer Models Initiative (HCMI), a collaborative effort from several institutions, is developing a biobank of PDOs from various cancer types, which will be accessible to the global scientific community (Bhatia et al., 2022; Aggarwal et al., 2023; Yao et al., 2020). The HCMI catalog currently includes more than 250 models derived from tumors at different stages of progression, from 28 primary sites, and with diverse mutational backgrounds. The characteristics of each model are available through the online tool^2^. All models in the HCMI collection are deposited at the american type culture collection (ATCC) and publicly available for research^3^. Another initiative, the Patient-Derived Models Repository (PDMR) led by the US National Cancer Institute (NCI), includes PDTX, *in vitro* patient-derived tumor cell cultures (PDC), cancer associated fibroblasts (CAF) and PDOs. The PDMR database currently includes over 400 PDO models, and their characteristics can be accessed on the project website^4^.

### 3.2 Personalized therapy and drug screening using PDO

**FIGURE 4:**
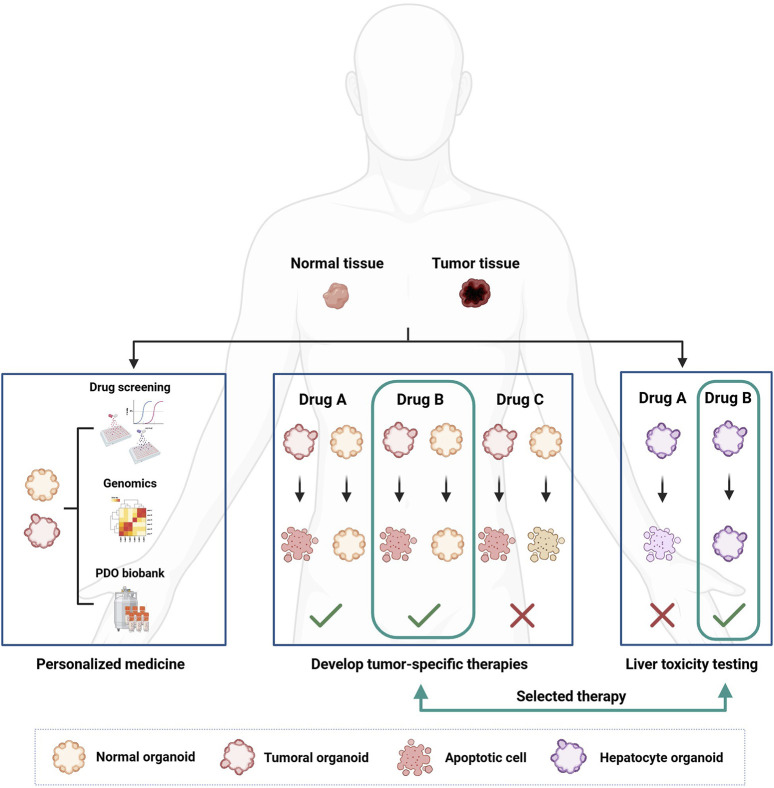
How organoids can be used for personalized cancer treatment and drug development. Organoids are developed from patient-derived tissue, both healthy and cancerous. Once developed, organoids can be characterized from a genetic point of view and used in drug screenings to correlate the genetic landscape of the tumor with the pharmacological response. The development of healthy organoids makes it possible to select less toxic drugs by searching for compounds that can selectively kill cancer cells only. Furthermore, organoids derived from healthy liver tissue can be used to test the hepatotoxicity of new drugs.

Since PDOs more accurately recapitulate the characteristics of the tumor of origin compared to other models (Sachs et al., 2018), they are considered suitable for identifying and testing new anticancer drugs (Figure 4). High-throughput drug screening methods using PDO technology are still under development (Mertens et al., 2023; Fatehullah et al., 2016; Jensen and Teng, 2020). However, small-scale screenings with PDOs have already shown promising results (Sachs et al., 2018; van de Wetering et al., 2015; Gao et al., 2014; He et al., 2023; Mertens et al., 2023; Yuan et al., 2022). For instance, a dual targeting treatment using mitogen-activated protein kinase (MEK) inhibitors led to cell growth arrest through cell cycle blockade in *Kras*-mutant CRC-PDOs, suggesting that combination therapies could offer a valid therapeutic option (Verissimo et al., 2016). Studies on PDOs obtained from CRC metastases have highlighted their ability to predict treatment response in a personalized manner. One study tested a library of compounds, some already in clinical use or in trials, on PDOs derived from metastatic CRC patients undergoing chemotherapy. Results showed that the PDO response to treatment predicted patient outcomes with 88% accuracy (Vlachogiannis et al., 2018). In another study, by Roy et al., PDOs were used to determine the most effective cytotoxic regimen for intraperitoneal chemotherapy in patients with CRC peritoneal metastases (Roy et al., 2017). Similarly, PDOs were used to determine the most effective treatment by evaluating current hyperthermic intraperitoneal chemotherapy (HIPEC) regimens on an individual patient basis (Ubink et al., 2019; Varinelli et al., 2024b).

PDOs derived from other tumor types have also been used in pharmacological screenings. For example, a study on PDOs from liver primary tumors demonstrated that inhibition of the MEK pathway may represent a new therapeutic approach for this tumor type (Vlachogiannis et al., 2018). Similarly, research on PDOs from prostate cancer revealed that E-cadherin (*CDH1*) gene deletions increase sensitivity to DNA damaging agents (Broutier et al., 2017; Shenoy et al., 2017). PDOs genetic profiling may help discover new epigenetic or genetic alterations that modulate drug response. Profiling data may also be used to stratify patients for more personalized treatments (Figures 3, 4), (Drost and Clevers, 2018; Gilazieva et al., 2020; Kim et al., 2022).

A unique feature of the PDO model is that it can be developed from both tumor and healthy tissues from the same patient, allowing drug selectivity for tumor cells to be assessed. This could help in the development of drugs with significantly lower toxicity than current treatments (Figure 4), (Calandrini and Drost, 2022; Kim et al., 2019; Crespo et al., 2017; Hennig et al., 2022; Gray et al., 2023). Drug-induced liver and heart toxicity are a major cause of failure in clinical trials (Ballet, 1997). Liver PDO biobanks could be used in preclinical testing to assess the hepatotoxicity of new compounds (Figure 4), (Katsuda et al., 2017; Shinozawa et al., 2021; Zhang C. J et al., 2023). Similarly, iPSC-derived cardiac PDO biobanks could be used to test cardiotoxicity induced by chemotherapeutic agents (Takasato et al., 2015; Chen et al., 2023).

Recent efforts have focused on establishing common criteria for developing, maintaining and testing PDOs to reduce variability between models. This will help generate more reproducible results that can be safely transferred into clinical applications (Lee et al., 2024).

## 4 Organoids and their role in basic cancer research

### 4.1 PDO for modeling tumorigenesis

Carcinogenesis is characterized by the accumulation of mutations in specific genes over time, which act as disease drivers (Stratton et al., 2009). Multiple mutational processes are active in neoplastic cells, making it difficult to study the pathogenesis associated with cancer-specific mutational signatures. In contrast, the genetic stability of healthy organoids offers an ideal platform to evaluate the association between particular mutational signatures and mutational processes, and identify the sequence of events that leads to cancer development (Behjati et al., 2014). For example, studies using organoids derived from different parts of the intestine and liver, capturing tissue heterogeneity, have shown that the high turnover of the intestinal crypt stem cells can promote mutagenic events induced by deamination processes. This leads to the acquisition of specific mutational signatures in CRC driver genes. Instead, mutations in the same driver genes arise through different mechanisms in hepatocellular carcinoma-derived PDOs (Blokzijl et al., 2016). This suggests that each organ may have specific mutational mechanisms that contribute to the accumulation of somatic mutations during the neoplastic process (S. Behjati et al., et al., 2014). The importance of understanding how cancer mutational signatures arise was recently demonstrated in breast cancer. Using mammary gland-derived PDOs, researchers showed that deficiencies in breast cancer gene 1 (*BRCA1*) and 2 (*BRCA2*) could be predicted from the tumor’s mutational signature. This finding has paved the way for selecting subgroups of patients who might benefit from treatments involving poly (ADP-ribose) polymerase (PARP) inhibitors (Davies et al., 2017). Similarly, Drost and colleagues studied the mutational consequences of DNA repair deficiency in healthy PDOs from normal colonic mucosa. They inactivated the DNA mismatch repair MutL homolog 1 (*MLH1*) and DNA glycosylase 1 (*NTHL1*) genes, respectively involved in mismatch repair (MMR) and base excision repair, and passaged the knock-out organoids (*MLH1* ^KO^ and *NTHL1* ^KO^) for two and 3 months to allow the mutations to accumulate. Whole-genome sequencing analysis revealed a higher number of base substitutions in both *MLH1* ^KO^ and *NTHL1* ^KO^ PDOs compared to normal organoids, with *MLH1* ^KO^ showing four times more substitutions. *MLH1* ^KO^ PDOs also presented an increased number of insertions or deletions, similar to those observed in MMR-deficient colorectal cancer. In contrast, *NTHL1* ^*KO*^ PDOs exhibited a non-random distribution of mutations similar to what was observed in normal cells (Drost et al., 2017).

The peculiarity of PDOs to present a stable genotype that preserves the original parental genetic landscape even after multiple passages (McGranahan and Swanton, 2017; Pauli et al., 2017), together with the possibility to generate PDOs from various regions of the same tissue sample, has provided key insight into cancer heterogeneity. Tumors are often genomically unstable, which contributes to intra-tumor heterogeneity (Drost and Clevers, 2018), playing a central role in cancer progression and the development of drug resistance. Despite its significance, the biological mechanisms behind this instability remain poorly understood (Greenman et al., 2007). PDOs derived from different tumor regions reveal varying mutational landscapes reflecting intra-tumor heterogeneity. This opens the door to therapeutic strategies tailored to patient-specific characteristics that can minimize or avoid chemotherapeutic resistance. For example, single-cell whole-genome sequencing of PDOs from rectal cancer revealed that radiotherapy resistance is driven by pre-existing radioresistant subclones, which either persist or expand, suggesting that radiation resistance can be predicted (Andel et al., 2024). Similarly, *ex vivo* chemotherapy screening with PDOs from pancreatic cancer, revealed patient-specific and intra-tumoral subclonal treatment sensitivities in patients who had disease progression (Le Compte et al., 2023). Karlsson’s group modeled occult preneoplasia in gastric cancer PDOs by biallelically inactivating the tumor protein TP53 gene (*TP53*), and growing clonally derived cultures for 2 years. Their study found that *TP53* loss led to progressive aneuploidy, with an apparent preferential order: initially, rare subclones with shared transcriptional programs achieved clonal dominance. These findings suggest that tumorigenesis in its early stages is predictable, and reveal evolutionary constraints and barriers to malignant transformation, with implications for early diagnosis and interception of aggressive and genomically unstable tumors (Karlsson et al., 2023).

Given that tumor heterogeneity profoundly affects drug response, PDO biobanks are now being designed to include multiple patient-derived samples, capturing regional heterogeneity and subclonal architecture. This approach has already been implemented for various cancers, including colorectal (Sasaki and Clevers, 2018), gastric (Yan et al., 2018), liver (Yang et al., 2024) and even glioblastoma (Jacob et al., 2020).

### 4.2 PDOs for modeling cancer progression using CRISPR-Cas9 technology

CRISPR-Cas9 (clustered regularly interspaced short palindromic repeats - CRISPR associated protein 9) gene editing allows for the precise introduction of mutations at specific genomic sites, enabling the development of PDO models to study the early stages of cancer progression (Matano et al., 2015). These models have revealed how neoplastic progression in CRC can drive tumor growth independently of the factors modulating the intestinal stem cell niche. Organoids derived from normal human intestinal epithelium, engineered with mutations in the tumor suppressor genes adenomatous polyposis coli (*APC*), SMAD family member 4 (*SMAD4*) and *TP53*, and in the oncogenes KRAS proto-oncogene, GTPase (*KRAS*) and/or phosphatidylinositol-4, 5-bisphosphate 3-kinase catalytic subunit alpha (*PIK3CA*), grew independently of niche factors *in vitro* and formed tumors upon implantation. However, these organoids could not colonize the liver, suggesting that while mutations in driver genes support stem cell growth and maintenance in the tumor microenvironment, they are insufficient to induce invasive behavior (Matano et al., 2015). Similarly, Fumagalli et al., dissected the adenoma-carcinoma sequence in an orthotopic PDO model derived from normal human colon, engineered with different CRC-associated mutations. They showed that the sequential accumulation of oncogenic mutations in *WNT*, *EGFR*, *TP53*, and transforming growth factor beta (*TGFB*) signaling pathways facilitates efficient tumor growth, migration, and metastatic colonization (Fumagalli et al., 2017).

Other studies have focused on specific genes critical for cancer development. Wang and colleagues used CRISPR-Cas9 to investigate the gain-of-function (GOF) and loss-of-function (LOF) effects of *TP53* in CRC. They found that removal of mutant *TP53* with GOF had no effect on proliferation or chemotherapeutic response, while restoring wild-type *TP53* halted PDO growth *in vitro*. This indicates that mutant *TP53* LOF effects, rather than GOF’s, are crucial for sustaining CRC growth (Wang et al., 2024). In another study on human primary gastric *TP53* ^*−/−*^ PDOs, knocking down the frequently mutated AT-rich interaction domain 1A (*ARID1A*) gene, led to a phenotype resembling *ARID1A*-mutant gastric cancers, particularly the MSI- (microsatellite instability) and (Epstein-Barr virus) EBV-associated subtypes, where *ARID1A* mutations are more common (Lo et al., 2021). Finally, PDOs from normal human cholangiocytes engineered with LOF mutations of the deubiquitinating enzyme BRCA1-associated protein 1 (*BAP1*) tumor suppressor gene, demonstrated that BAP1 regulates chromatin accessibility, and controls the expression of cell junction and cytoskeleton components, essential to maintain epithelial characteristics. The introduction of mutations in genes *TP53*, *SMAD4*, phosphatase and tensin homolog (*PTEN*) and neurofibromin 1 (*NF1*), commonly altered in cholangiocarcinoma, resulted in the acquisition of malignant features upon xenotransplantation. These findings suggest that BAP1’s role in regulating epithelial cell identity through chromatin accessibility is key to its tumor suppressor function (Artegiani et al., 2019).

CRISPR-Cas9 technology has also been used to study cancer-related signaling pathways on PDOs. For example, the response to TGFB stimulation in genetically diverse CRC precursor lesions, was investigated in tubular adenoma (TAd) and sessile serrated adenoma (SSA) organoids. TAd organoids, which progresses to the chromosomally unstable CRC subtype, were generated from intestinal tissue of subjects carrying an *APC* inactivating mutation. Instead, SSA organoids, which can progress to the mesenchymal phenotype with poor prognosis, were obtained by engineering PDOs from normal colon tissue to carry the B-Raf proto-oncogene, serine/threonine kinase (*BRAF*) V600E mutation. TGFB treatment induced apoptosis in TAd-PDOs, but led to development of a mesenchymal phenotype in SSA-PDOs (Fessler et al., 2016).

CRISPR-Cas9 technology has significantly enhanced the ability to perform rapid and effective genetic and epigenetic screenings in PDOs. For instance, a genome-wide editing approach in pancreatic cancer PDOs was used to repair specific mutations in oncogenes, reversing the tumor phenotype. This led to the identification of genes involved in modulating the response to gemcitabine, a pancreatic cancer drug. Through genome editing PDOs with various genetic backgrounds, researchers identified genes associated with resistance, such as the deoxycytidine kinase (*DCK*), and with sensitivity, such as checkpoint kinase 1 (*CHEK1*), HUS1 checkpoint clamp component (*HUS1*) and RAD1 checkpoint DNA exonuclease (*RAD1*) (Ubhi et al., 2024). Considering that resistance to gemcitabine occurs after only a few rounds of treatment, the identification of these genes is of great importance and may help improve the efficacy of the therapy. In another study, Michels et al. developed a platform for pooled CRISPR-Cas9 screening in colon PDOs with *APC* ^−/−^ and *KRASG12D* mutations. The authors screened a pan-cancer tumor suppressor gene library using TGFB sensitivity as a phenotypic trait allowing for robust positive selection, and identified the transforming growth factor beta receptor 2 receptor (*TGFBR2*) gene as a key mediator of CRC growth (Michels et al., 2020). Similarly, in breast PDOs, knocking out the tumor suppressor genes *TP53*, *PTEN*, RB transcriptional corepressor 1 (*RB1*) and *NF1*, led to the development of estrogen receptor-positive luminal tumors upon transplantation in mice, which responded to endocrine therapy and chemotherapy (Dekkers et al., 2020).

These examples demonstrate how the combination of PDO technology and CRISPR-Cas9 provides an invaluable platform for studying the mechanistic role of cancer genes in a biologically relevant human context.

## 5 Current limitations and challenging in PDO technology

Despite their significant potential in cancer research PDO models face several challenges, including inability to fully recapitulate tumor heterogeneity, lack of standardization and difficulty in replicating complex tissue structures and functions.

Tumors consist of diverse cellular subpopulations with distinct genetic mutations and phenotypic traits, which often display differences in proliferation rates, invasive potential, and response to therapy. PDO cultures tend to selectively expand certain subpopulations, leading to the loss of other cellular variants present in the original tumor. This issue is especially evident in highly heterogeneous cancers, such as triple-negative breast cancer and glioblastoma, where conventional PDO protocols may preferentially expand stem-like populations with high proliferative capacity, suppressing the maintenance of other clones *in vitro* (Skala et al., 2022; Mathur, 2024). The *in vitro* environment may also introduce selective pressure that favors the growth of specific clones, potentially leading to an artificial evolution that does not reflect tumor *in vivo* dynamics. Furthermore, certain driver mutations may become overrepresented, while subclonal populations critical for therapy resistance might be lost or underrepresented (Skala et al., 2022; Mathur, 2024). Tumor heterogeneity also varies significantly between patients within the same cancer type. Even within the same histological classification, tumors can exhibit considerable differences in cellular composition, mutational landscapes and drug responses (Skala et al., 2022; Mathur, 2024). By preferentially selecting specific subpopulations, the current process of PDO generation may also limit the ability of PDO models to capture interpatient tumoral heterogeneity.

Another significant challenge is the lack of standardized protocols and the absence of an international consortium of PDO specialists working to establish reproducible, validated methodologies. This leads to considerable variability in results across laboratories, which hampers reproducibility in large-scale studies and clinical applications. Variations in culture media, growth factors sources, and splitting techniques can significantly impact the expansion of specific cell subsets, undermining the reliability of experimental outcomes.

Tumors do not exist in isolation but within a complex TME, which includes CAFs, immune cells, blood vessels, and the ECM. These components actively regulate tumor growth, progression, and response to therapies (Klein, 2020). This dynamic environment is crucial for understanding therapy resistance and tumor evolution, but is often underrepresented in standard PDO cultures. For instance, CAFs contribute to ECM stiffness, pro-tumor cytokine secretion and immune modulation (Yang et al., 2023), and display pro-tumor activities. Also, the immune system plays a critical role in tumor progression and response to immunotherapy (Binnewies et al., 2018). However, conventional PDO models lack stromal components, limiting their ability to replicate tumor-stroma interactions. Similarly, PDOs are typically cultured in immunodeficient conditions, making them unsuitable for studying immune checkpoint therapies. Tumor angiogenesis, which supports growth and affects drug and nutrient accessibility, is another key feature that current PDO protocols fail to recreate, limiting the modeling of tumor hypoxia, a major factor in therapy resistance (Suvac et al., 2025). These limitations significantly reduce the physiological relevance and translational potential of PDO models in preclinical oncology research.

PDO biobanks, a promising tool for precision oncology, enable patient-specific drug screening and contribute to advancing cancer research (Drost and Clevers, 2018). However, their large-scale implementation faces several challenges. Establishing PDO cultures is time-consuming and resource-intensive, often requiring extended periods to develop stable cultures (Tong et al., 2024). Each step, from tissue dissociation to *in vitro* expansion, requires careful optimization to ensure cellular viability and accurate tumor representation. Additionally, interpatient variability impacts PDO success rate, with some tumor histotypes being easier to culture into organoids, while others fail, limiting biobank potential (Zhao et al., 2022). Developing PDO biobanks also requires specialized infrastructure and personnel, as PDOs are typically cultured in costly, in-house made growth-factor-enriched media that are prone to batch-to-batch variability (Bose et al., 2021). Moreover, the lack of standardized protocols for PDO development remains a significant barrier, as variations in culture conditions and techniques across labs, can lead to inconsistent PDO behavior, hindering reproducibility and limiting their utility in large-scale drug screenings (Andrews and Kriegstein, 2022). Another concern is genetic and phenotypic drift during long-term culture, which may affect the clinical relevance of PDOs in biobanks (Andrews and Kriegstein, 2022). Cryopreservation also poses a challenge, as do ethical and regulatory issues like privacy regulations and informed consent, which complicate the use of PDOs in drug screening and precision medicine (Xie et al., 2023).

Despite these challenges, organoid technology has made significant progress. However, accurately modeling tumor heterogeneity and the TME remains a key obstacle. To enhance the translational relevance of PDOs in precision oncology, innovative approaches that integrate microenvironmental complexity, genetic diversity, and strategies for improving reproducibility are essential (Figure 1). Co-culture techniques with stromal cells, immune cells, and other TME components could help PDOs better reflect *in vivo* tumor features. Moreover, microfluidic platforms and organ-on-a-chip systems may provide more precise simulations of TME interactions, improving model reproducibility and fidelity. To preserve tumor heterogeneity, genetic engineering and epigenetic modulation strategies such as CRISPR/Cas9 and epigenetic modifiers could help maintain a more faithful representation of tumor heterogeneity within PDOs (Figure 1). As for PDO-based biobanks, improving cryopreservation methods to maintain both viability and genomic stability over time will be crucial for large-scale biobanks and personalized therapies.

Standardizing culture protocols and analytical methods across research centers could reduce variability, lower costs, and streamline PDO generation, making them more accessible for clinical and preclinical use. Addressing these challenges will be key to fully unlocking the potential of PDOs in oncology research and precision medicine.

## 6 Advancing cancer models by incorporating the tumor microenvironment: focus on the extracellular matrix

**FIGURE 5:**
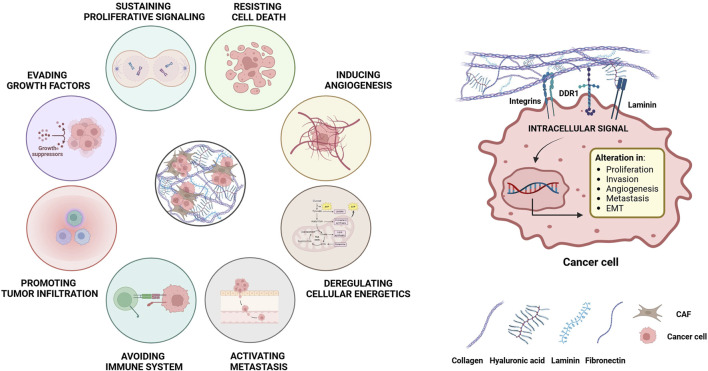
Modulation of the main hallmarks of cancer by the ECM. The remodeling of the ECM during cancer development allows the formation of bonds between the proteins and the molecules that make up the matrix, such as collagen, fibronectin and laminin with different receptors on the cell surface. These interactions activate intracellular signaling pathways that promote key pro-tumor actions, including survival, proliferation, angiogenesis, metastasis and resistance to chemotherapy. Both neoplastic cells and the non-malignant stromal cells contribute to those processes and, in turn, are influenced by changes in the matrix. These modifications encompass i) biochemical changes, ii) secretion of specific growth factors, iii) alterations in matrix hydration, iv) post-translational modifications, v) changes in biomechanical properties, vi) massive collagen deposition leading to a more fibrotic state, vii) alterations in the structural organization with changes in ECM porosity, viii) deregulated turnover rates of matrix components and ix) disruption of cell-cell adhesion interactions due to expression of binding proteins.

Cancer arises from the accumulation of mutations in specific genes over time, combined with complex molecular interactions between tumor cells and their microenvironment, which is influenced by paracrine cell-cell communication, where neoplastic cells secrete specific factors (Figure 5), (de Visser and Joyce, 2023; Najafi et al., 2019; Lorenc et al., 2023; Buruiană et al., 2024; Popova and Jücker, 2022). Significant advances in understanding tumorigenesis have improved our knowledge of cancer biology and enhanced patient survival, largely through improved surgical techniques, chemotherapy regimens, and the introduction of immunotherapy (Steinbach, 2024; Steeg, 2016; Riley et al., 2019; Yuki et al., 2020; Neal et al., 2018; Alsaed et al., 2024). Despite the advancement, cancer remains complex and requires deeper exploration, especially metastatic disease, as the biological mechanisms driving the colonization of metastatic sites by circulating cells remain unclear (Peinado et al., 2017; Ceelen et al., 2020; Xue et al., 2020). Recently, there has been growing recognition that the neoplastic cells operate within a dynamic microenvironment, crucial to both cancer development and metastasis, including the formation of the metastatic niche (Rabas et al., 2024; Zhang et al., 2024; Zhang et al., 2020; Peinado et al., 2017; Akhtar et al., 2019). The extracellular matrix (ECM), traditionally considered a passive scaffold, is now recognised as an active metastasis promoter in target organs, making it a focus of recent studies (Figure 5), (Henke et al., 2020; Panciera et al., 2020; Cox, 2021; Winkler et al., 2020; Reuten et al., 2021; Sleeboom et al., 2024). Given the significant remodeling of the ECM and its central role in both primary tumors and metastases, future studies into its precise function could lead to new insights and treatment strategies.

### 6.1 The extracellular matrix

The ECM is an acellular component found in all tissues, composed of molecules secreted and assembled into insoluble structures that are critical for organ development, maintenance and repair of damaged tissue. The ECM provides structural and mechanical support for resident cells, and plays a pivotal role in regulating cell proliferation, survival, migration and invasion (Cox, 2021). The ECM is made up of hundreds of different proteins, interacting to form a complex 3D architecture. Due to the numerous post-translational modifications and specific transcripts encoding matrix protein variants, the human body can produce a virtually limitless variety of ECM components (Yamada et al., 2022). Moreover, the ECM is dynamic, undergoing continuous remodeling in response to both external and internal stimuli (Wang et al., 2022).

There is a reciprocal interaction between cells and the ECM, involving processes such as cell deposition along the 3D matrix architecture, selective matrix remodeling, and the modulation of cellular functions by the ECM. These complex and reciprocal interactions have been termed “dynamic reciprocity,” and highlight the importance of the ECM in tissue and organ physiology (Cox and Erler, 2014). The ECM undergoes significant deregulation in cancer and actively participates in tumorigenesis, although it can also exhibit some anti-tumor properties (Figure 5), (Yuan et al., 2023). Tumor desmoplasia, a common feature of several solid tumors, often resembles tissue fibrosis (Cox and Erler, 2014), highlighting how tumor development is accompanied by matrix remodelling. Indeed, the loss of proper ECM organization is now considered a key hallmark of cancer development. It is crucial to understand how both cancer and non-malignant stromal cells contribute to, and are affected by ECM deposition and remodeling during cancer (Figure 5), (Winkler et al., 2020). Over the past threedecades, interest in the role of ECM in cancer has surged, and the ECM has been identified as a prognostic and diagnostic biomarker and even a potential therapeutic target for various solid tumors (Cox, 2021).

### 6.2 Changes in the ECM during cancer

**FIGURE 6:**
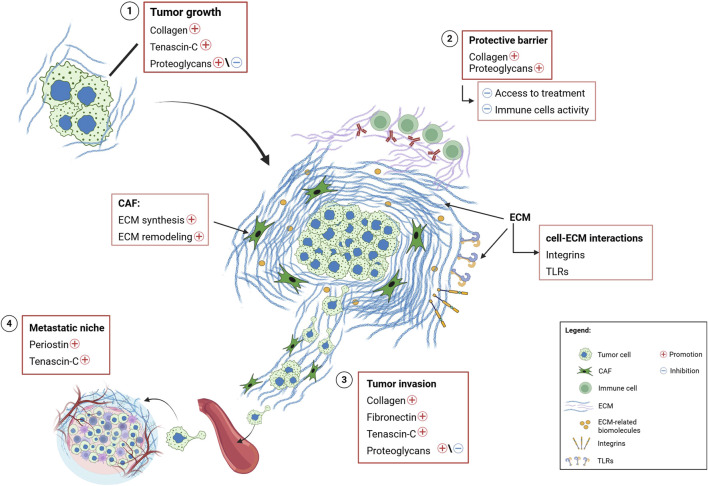
Schematic illustration of the role and functions of extracellular matrix in the tumor microenvironment. Summary of the main effects and key cell-matrix interactions involving ECM molecules in various types of cancer. **1)** Biochemical and biophysical interactions, mediated by cell-ECM communication through specific receptors on the ECM (e.g., integrins), promote the expression of collagen, tenascin-c and, in some cases, proteoglycans, favoring tumor growth. **2)** ECM remodeling results in an accumulation of structural proteins (such as collagen and proteoglycans), at the tumor site, forming a protective barrier against drugs and inhibiting immune cells activity. Additionally, ECM remodeling activates signaling pathways that regulate cell-ECM interaction *via* specific receptors (i.e., integrins and Toll-like receptors). **3)** The overexpression of structural and ECM-related proteins enhances tumor invasion. **4)** The overexpression of periostin and tenascin-c, driven by remodeling activity of neoplastic cells and the TME, contributes to the formation of the metastatic niche. Legend, ECM, Extracellular matrix; CAF, Cancer-Associated Fibroblast; TLRs, Toll-like Receptors; TME, Tumor Microenvironment.

Many solid tumors are characterized by high levels of fibrosis, with recent studies showing that the ECM at tumor sites is primarily produced by cells in the stromal microenvironment, particularly CAFs (Figure 6), (Liu et al., 2019). A variety of CAF subtypes have been described across different cancer types, although their exact origin remains unclear (Pereira et al., 2019). Depending on their tissue localization, these CAF subtypes secrete specific molecules that induce fibrosis, contributing to ECM remodeling and explaining in part the high matrix heterogeneity observed in cancer (Figure 6), (Zhang H. et al., 2023). CAFs are resident fibroblasts activated by growing tumor cells. In turn, these activated CAFs influence other stromal cells, like adipocytes and mesothelial cells, creating a complex network that remodels the ECM, promoting tumor development (Kalluri, 2016). CAFs are the primary source of ECM deposition in cancer, with many matrix components being fibrotic-like molecules (Tian et al., 2019). Indeed, CAFs have been linked to poor prognosis in several solid tumors (Liu et al., 2016).

Exosomes, membrane-bound extracellular vesicles secreted from the endosomal compartment, play a significant role in modulating CAFs and other stromal cells. They facilitate cross-talk between tumor cells, and between cells and their microenvironment, by transporting factors like TGFB, which can reprogram stromal cells (Webber et al., 2015). Additionally, CAFs have a dynamic phenotype that can be influenced by growth factors and cytokines, such as TGFB and interleukin-1 (IL1), through the RAS proto-oncogene GTPase (RAS) and signal transducer and activator of transcription (STAT) pathways (Vasiukov et al., 2020). The spatial and temporal distribution of factors released by CAFs influences ECM composition. Rather than eliminating CAFs and other stromal cells, reprogramming them may offer a promising therapeutic strategy (Cox, 2021).

## 7 ECM and its involvement in cellular signaling

### 7.1 The role of growth factors in ECM-mediated signaling

The ECM strongly influences intracellular signaling both directly and indirectly, by creating a complex network of interactions that leads to cross-regulation between various signaling pathways (Figures 5, 6). For instance, integrin-mediated signaling is significantly affected by ECM stiffness, which enhances their activity by promoting the activation of tyrosine-kinase receptors such as EGFR, HER2, vascular endothelial growth factor receptor (VEGFR) and hepatocyte growth factor receptor (HGFR), especially in cancer cells (Guo et al., 2006). For example, ECM stiffness activates HER2 signaling, leading to increased resistance to standard therapies (Weigelt et al., 2010). While EGF-mediated signaling is driven in certain tumors by mechanosensation, the process by which cells sense and respond to physical forces in their environment through ECM remodeling (Grasset et al., 2018). ECM stiffness also modulates the activation of pathways mediated by mitogen-activated protein kinase (MAPK)- and yes1 associated transcriptional regulator (YAP)- tafazzin (TAZ) (YAP/TAZ), both of which are associated with the onset of chemoresistance (Nguyen and Yi, 2019). Additionally, the rho associated coiled-coil containing protein kinase (ROCK) pathway has been identified as an important player in ECM modulation and in the response to matrix remodeling stimuli in both tumor cells and CAFs (Vennin et al., 2017).

### 7.2 Integrins function as ECM receptors

Integrins are heterodimeric cell surface receptors that serve as primary mediators of cell-ECM communication which can activate multiple signaling pathways (Kechagia et al., 2019). The expression of specific integrins involved in tumor ECM remodeling is critical during neoplastic development (Figure 6). Different integrins are overexpressed in different tumor types and typically facilitate cell invasion and metastasis through the formation of matrix-dependent junctions (Hamidi and Ivaska, 2018). Certain integrin heterodimers, found in specific regions of the ECM, promote cell survival and contribute to the development of resistance to therapeutic treatments (Madamanchi et al., 2014). For example, the fibronectin-binding integrin ανβ3 heterodimer is associated with tumor cell survival during tumor development (Young et al., 2020). In addition, overexpression of the α5β1 dimer activates CAFs, promoting tumor fibrosis, which acts as a protective barrier against treatment penetration (Kuninty et al., 2019). The role of integrins in cancer development depends on both the cell type in which they are expressed and the biochemical properties of the ECM. During the epithelial-mesenchymal transition (EMT), a switch from E-cadherin to N-cadherin is observed, along with a transition from the α6β4 dimer to β1 and β3 integrin heterodimers (Janiszewska et al., 2020). This switch enables cancer cells to pass from a cell-cell to a cell-ECM attachment type, promoting adhesion to type I collagen fibers and facilitating neoplastic invasion (Wheelock et al., 2008). In summary, the adhesion complexes formed by integrins and cadherins create an intricate network that influences the cell cytoskeleton and mediates interactions between cells and the ECM. This network allows cancer cells to respond to stress signals, triggering biochemical and biomechanical processes that affect the surrounding TME (Figure 6), (Zuidema et al., 2020).

### 7.3 Other non-integrin ECM-binding receptors and their functions

Beyond integrins, the ECM binds other receptors, such as discoidin domain-containing receptors 1 (DDR1) and 2 (DDR2), osteoclast-associated immunoglobulin-like receptor (OSCAR), syndecans, urokinase-type plasminogen activator receptor-associated protein (UPARAP), and leukocyte-associated immunoglobulin-like receptor 1 (LAIR1). These receptors activate downstream signaling pathways in response to interactions between the extracellular and intracellular environments (Cox, 2021). In the context of tumors, activation of DDR1 and DDR2 in CAFs increases ECM stiffness, and promotes the deposition of a new matrix that facilitates metastatic dissemination and impairs the response to chemotherapy (Vh Bayer, S. et al., 2019). Syndecan-4, which is often deregulated in solid tumors, modulates intracellular signaling in response to localized tension between cells and the ECM. This process involves mechanochemical signaling through the activation of membrane bound EGFR, integrin β1, and the intracellular transcription coactivator YAP (Chronopoulos et al., 2020).

## 8 Biomechanical properties and functions of the ECM in cancer

Mechanobiology combines biology, physics, chemistry, and engineering to study the mechanical properties of cells and tissues, as well as the interactions between proteins, cells and the microenvironment (Holuigue et al., 2022). These processes play a crucial role in the biological mechanisms that drive tissue development, such as the regulation of cell polarity, gene expression and stem cells differentiation (Smith et al., 2018). The biomechanical properties of the tumor ECM significantly influence the behavior of neoplastic cells, CAFs, and various stromal and immune cells, highlighting how mechano-modulation affects several key aspects of cancer (Hoffmann and Ponik, 2020). The ECM exhibits complex mechanical properties (Hoffmann and Ponik, 2020), such as viscoelasticity, mechanical and nonlinear plasticity. Due to this complexity, cellular responses influenced by the ECM are regulated on specific timeframes and are bidirectional (Gong et al., 2018). Key signaling pathways, such as focal adhesion kinase (FAK)-SRC proto-oncogene, non-receptor tyrosine kinase (SRC) (FAK/SRC), ERK, YAP/TAZ and ROCK, mediate these biomechanical functions (Pratt et al., 2020). These pathways are often dysregulated in cancer, contributing to resistance, immune escape, enhanced invasion, survival and cell proliferation. Thus, both the morphological and mechanical properties of the ECM play crucial roles in regulating asymmetric stem cell division and differentiation, epithelial-mesenchymal transition, cell migration, and neoplastic cell differentiation and proliferation, both in primary and metastatic sites (Cox, 2021). Moreover, tumor induced ECM stiffening of the tumor-surrounding tissues directly contributes to metastatic spread by creating a more favorable microenvironment for neoplastic cells, which can also promote tumor development and drug resistance (Cox, 2021).

### 8.1 The atomic force microscopy for studying biomechanical properties

The mechanical properties of 3D models, such as PDOs, spheroids or scaffold-based systems, can be precisely adjusted to simulate a wide range of tissue stiffnesses (McKenzie et al., 2018). Mechanobiological applications have been integrated into clinical and biological studies, demonstrating their potential for diagnosing various diseases and providing deeper insights into physiological and pathological processes, including cell-cell and cell-microenvironment interactions (Stylianou et al., 2018; Holuigue et al., 2022; Alcaraz et al., 2018; Zemła et al., 2018a).

The development of atomic force microscopy (AFM) in recent decades enabled the study of ECM stiffness at the nanoscale. This non-optical imaging technique enables precise and non-destructive measurement of the surface topography of samples with very high resolution in air, liquids or ultra-high vacuum conditions (Alessandrini and Facci, 2005). The nanoindentation method, commonly used in AFM experiments, involves a hard tip pressing against the sample surface until deformation occurs. The relationship between the applied force and surface deformation provides insights into the local mechanical properties of the sample, including its hardness and Young’s modulus, which measure tensile stiffness (Zemła et al., 2020; Holuigue et al., 2023; Chighizola et al., 2021). AFM can be applied to a wide range of specimens from tissues to subcellular biomolecules (Chighizola et al., 2020; Tian et al., 2015; Zemła et al., 2018b; Graham et al., 2010; Plodinec and Lim, 2015). It is adaptable to more advanced biological models, including spheroids and organoids (Holuigue et al., 2023; Paradiso et al., 2021) and has been widely used in both clinical and basic research, highlighting its potential in diagnosing diseases, including cancer, and improving patient care (Stylianou et al., 2018; Lorenc et al., 2023; Kubiak et al., 2020).

## 9 The role of ECM in the development of metastases

Tumor cells need to acquire several specific properties to metastasize, including increased motility, invasiveness, plasticity, and the ability to modulate and colonize distant metastatic sites while altering the local microenvironment (Cox, 2021). The ECM plays a crucial role in regulating all these processes, making it a key player in metastasis development (Figures 5, 6). Tumors often have thickened collagen fibers arranged linearly and radially from the tumor mass. This pattern facilitates metastasis by acting as a “highway” for tumor cells, facilitating their migration from the primary site (Conklin et al., 2018). Type I collagen is the main protein involved in this process in both primary and metastatic tumor sites, with matrix metalloproteinases (MMPs) further supporting the invasion of circulating tumor cells (Feinberg et al., 2018). The tumor ECM also promotes metastasis actively. For example, the upregulation of collagen I-activated tetraspanin (*TM4SF1*), activates a non-canonical DDR1-mediated pathway that induces cell growth and enhances stem and metastatic traits in various cancers (Gao et al., 2016).

During metastatic progression, matrix remodeling triggers mechanisms that facilitate the formation of “invadopodia,” actin-rich plasma membrane protrusions that interact with integrin receptors and form focal adhesions. This process enables tumor invasion after the local ECM has been degraded by MMPs (Cox, 2021). The increased stiffness of the ECM contributes to this process by influencing the assembly of focal adhesions (Parekh et al., 2011). When the physical barriers around the tumor are breached, neoplastic cell migration is initiated (Figure 6). This process is mediated by ras homolog family members (Rho) and rac family small Rac (Rac) GTPases (Parri and & Chiarugi, 2010; Sanz-Moreno et al., 2008). Constitutively activated Ras stimulates Rho activity, promoting a phenotype that induces amoeboid-like migration, while the absence of the tumor suppressor protein p53 prevents the inhibition of cell migration (Xia and Land, 2007). Once metastatic cells reach the target organ parenchyma, they must navigate through the tissue vasculature to extravasate and colonize secondary sites (Celià-Terrassa et al., 2012). In this process, the metastatic potential of transformed cells is enhanced by the EMT switch, which is induced by local ECM degradation or by high TGFB levels secreted by infiltrating immune cells (Pickup et al., 2014). An increase in ECM stiffness promotes TGFB-induced EMT, fostering a basal-like phenotype of tumor cells and stimulating metastatic spread (Leight et al., 2012).

## 10 Decellularized ECM for building advanced *ex vivo* cancer models

Alterations in the ECM and the microenvironment are characteristic of cancer and contribute to chemotherapy resistance (Ferreira et al., 2020). As the tumor develops, mutations accumulate in cancer cells and the deregulation of stromal cells leads to both biomechanical and functional changes in the ECM (Kalluri, 2016). These changes are associated with increased cancer cell invasiveness and resistance to therapy, which often correlates with poor prognosis (Figures 5, 6), (Hamidi and Ivaska, 2018; Brabletz et al., 2018). Therefore, it is crucial for an accurate cancer model to incorporate ECM or its main components, as they play essential roles in cancer progression and treatment resistance. This has sparked growing interest in creating ECMs that can be used as scaffolds for the growth of tumor cells. However, replicating ECMs that mimic those found in solid tumors remains challenging, due to the complexity and dynamic nature of the tumor microenvironment (Ferreira et al., 2020). The development of ECMs derived from cells or tissues offers a promising solution, as they better reproduce the tissue microenvironment and promote relevant cellular interactions while maintaining biocompatibility and degradability. Scaffolds with these characteristics can be produced by stromal cells, such as fibroblasts, cultured under specific conditions (Sensi et al., 2018). Alternatively, ECM-based scaffolds can be obtained directly from tissue, by removing cells. This process, referred to as decellularization, generates decellularized ECMs (dECMs) that preserve the natural mechanical integrity of the matrix.

### 10.1 Decellularization methods

**FIGURE 7:**
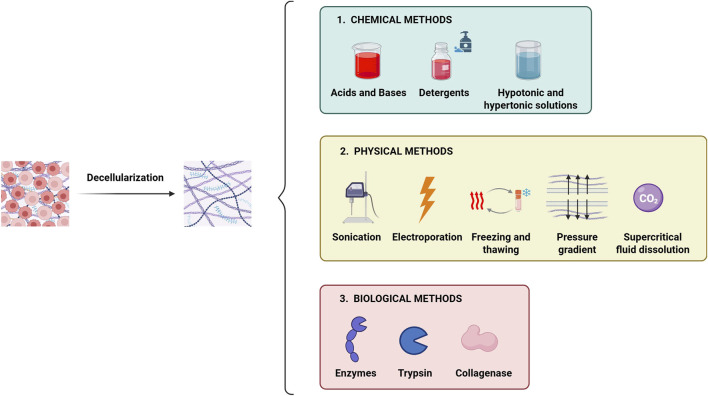
Main methods used for tissue decellularization. The decellularization process involves the complete removal of the cellular component from the tissue while preserving the native ECM micro-architecture and biochemical properties. The main decellularization methods are categorized into: i) chemical type, ii) physical type and iii) biological type. Often, combining different methods enhances the efficiency of decellularization.

To effectively decellularize tissues, the cells must be degraded while preserving the ECM’s microarchitecture, biochemical composition and bioactivity (Gilpin and Yang, 2017). These decellularizing methods can be physical, enzymatic and chemical (Figure 7). Depending on the method and the tissue thickness, decellularization can take from minutes to 72 h (Maia et al., 2020). After the process, dECMs are sterilized using peracetic acid, ethanol, antibiotics and ultraviolet or gamma radiation (Ferreira et al., 2020), and their biochemical properties, such as collagen content and the absence of DNA and RNA, are assessed to confirm decellularization (Koh et al., 2018).

### 10.2 Physical decellularization

Physical methods for decellularization include magnetic stirring, sonication, high hydrostatic pressure and freeze-thawing procedures aimed at disrupting cell membranes (Gilpin and Yang, 2017; Sun et al., 2018). These techniques are usually combined with frequent washing steps, followed by the use of chemical solutions (Figure 7), (Gilpin and Yang, 2017). To date, the use of pressure gradients has emerged as the most promising method for physical decellularization. Hydrostatic pressure, especially when used with chemical agents, has proven effective in increasing cell lysis and removing cell debris (Watanabe et al., 2019). Another promising method is the supercritical carbon dioxide (CO_2_)-based decellularization, which may better preserve the native composition of the ECM (Matthews et al., 2018).

### 10.3 Chemical decellularization

Chemical decellularization involves using acids, bases, hypertonic solutions and detergents to break down cell membranes and eliminate debris (Figure 7). Acids like acetic acid and peracetic acid, as well as bases like ammonium hydroxide, enhance decellularization (Hasmad et al., 2018). However, bases are generally used only in the early stages of the process, especially for very dense samples, due to their pronounced aggressiveness (Scherzer et al., 2015). Still, both acidic and basic solutions can degrade ECM components, altering the structural stability of the matrix itself (Hasmad et al., 2018). Thus, milder detergents are often preferred to maintain the ECM’s structural integrity (Ferreira et al., 2020).

### 10.4 Detergent-based and enzymatic decellularization

Detergent based decellularization methods are classified as ionic, nonionic (based on polyoxymethylene or a glycoside) or zwitterionic (they carry one positively and one negatively charged group resulting in no net charge, like non-ionic surfactants). Non-ionic detergents, such as Triton-X100, are considered gentle detergents, preserving the native structure of proteins and their enzymatic activity. Ionic detergents, such as sodium dodecyl sulfate (SDS) and Triton-X200, have a high decellularization capacity but are more aggressive (Simsa et al., 2018). In tissues with high lipid content, detergent-based decellularization methods are often combined with solvents such as methanol, ethanol or chloroform to deplete lipids (Figure 7), (Fairfield et al., 2019).

Enzymatic decellularization relies on specific enzymes, such as trypsin, peptidases or nucleases, to digest and remove cells. These methods are typically combined with physical and chemical techniques to ensure complete removal of genetic material (Figure 7), (Taylor et al., 2018). This combination allows for proper preservation of the architecture and protein composition of complex tissues (Scanlon et al., 2013), (Figure 5).

## 11 dECMs for *in vitro* tumor modelling

**FIGURE 8:**
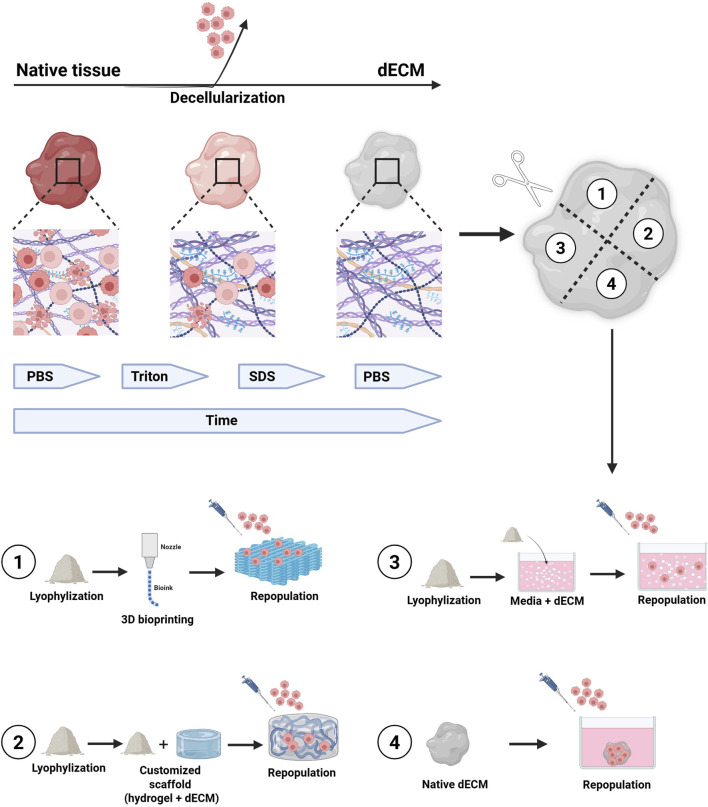
The figure illustrates the general process for obtaining decellularized tissues to be used as scaffolds for organoid culture and/or different cellular subpopulations. The tissue undergoes several washing cycles with non-ionic detergents, until the complete removal of cellular material from the native tissue. The decellularization process can take from hours to days, depending on the tissue’s size and origin. Once decellularized, the tissue can be used in its lyophilized and naïve form for the culture of organoids and/or other cell types within the microenvironment. **1)** lyophilized dECM can be used to produce biocompatible inks for 3D bioprinting of scaffolds, which can then be repopulated with organoids. 2) Lyophilized dECM can be resuspended at various concentrations in synthetic hydrogels and used as support matrices for organoid growth as an alternative to Matrigel. **3)** Lyophilized dECM can be resuspended, at different concentrations, directly inside the organoid culture medium. **4)** Naïve dECM can be used directly as scaffolds and repopulated with organoids. Legend: dECM, decellularized Extracellular Matrix; PBS, Phosphate Buffer Saline; SDS, Sodium Dodecyl Sulfate.

Decellularized matrices serve as scaffolds for the growth of normal and neoplastic cells, and repopulated dECMs provide an *ex vivo* model of the disease. These models offer several advantages, such as the preservation of complex architectural structures, ease of handling and analysis, and relevant biological properties (Figure 8). Recent studies have increasingly employed dECMs to create 3D tumor models *in vitro* (Rijal and Li, 2017; Koh et al., 2018; Sun et al., 2018; Tian et al., 2018; Milton et al., 2024; D'Angelo et al., 2020; Grey et al., 2018; Miyauchi et al., 2017; Sensi et al., 2018), highlighting their ability to replicate the critical interactions between tumor cells and the ECM. These interactions are essential for reproducing key cancer processes, including tumor initiation, progression and metastasis (Ferreira et al., 2020).

### 11.1 *In vitro* tumor models based on repopulated dECMs

**FIGURE 9:**
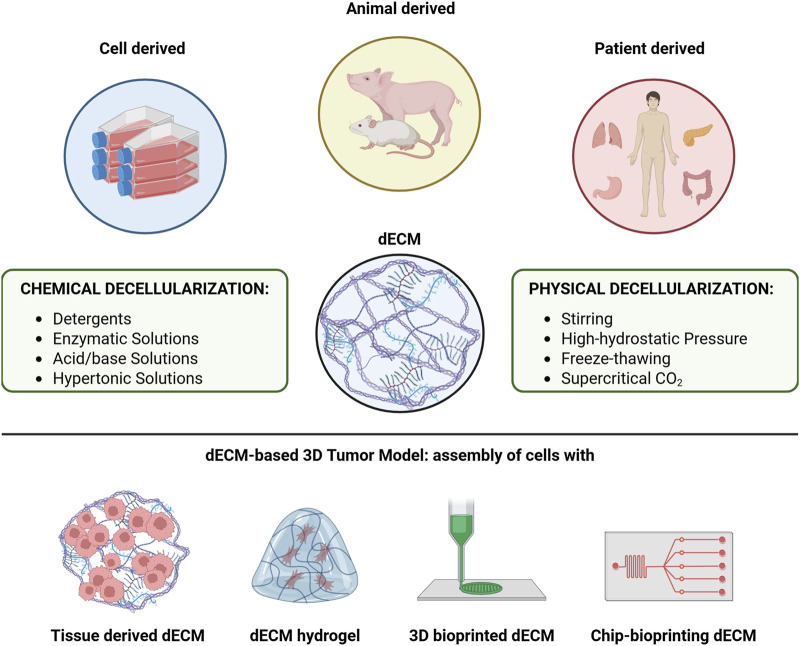
Overview of the main sources from which dECMs are obtained and of the main methodologies used to obtain them, including the various modelling strategies to develop 3D disease models that can summarize the main features of a tumor.

Tumor models created with tissue-derived dECMs repopulated by cancer cells retain a fibrous microarchitecture closely resembling that of the original tissue (Keeton et al., 2018; Varinelli et al., 2023). These models also maintain the ECM’s biomolecular composition, including growth factors, structural proteins and cytokines/chemokines, which support neoplastic cells and influence their behavior (Genovese et al., 2014), effectively replicating the interaction networks involved in tumor growth, invasion and metastasis (Brabletz et al., 2018). Furthermore, dECM-based models can be constructed from tumors at various stages of malignant progression, reproducing the complexity and spatio-temporal nature of cancer (Figures 8, 9), (Scanlon et al., 2013).

Despite these advantages, current dECM repopulation techniques are based on the direct seeding of a specific cell subtype on the surface of a dECM, resulting in a random cell distribution, limiting the ability to target specific regions within the matrix. Researchers are exploring methods such as micromanipulation, to inject cells at defined sites, which could improve repopulation efficiency and enable a more precise study of cell-ECM interactions (Figures 8, 9).

### 11.2 *In vitro* tumor models using dECM-based hydrogels

**FIGURE 10:**
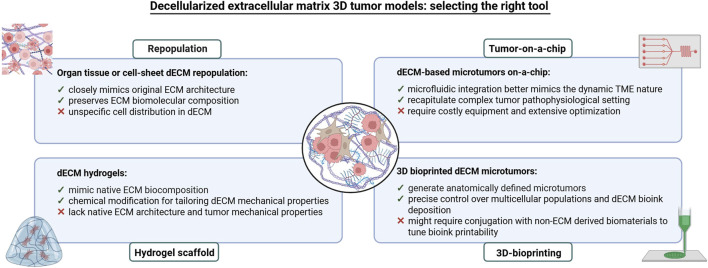
Overview of the main strategies used to develop 3D *in vitro* tumor models based on dECM. The main advantages and limitations of all strategies are also indicated.

dECM models can also be created using hydrogels, polymeric materials that mimic the native ECM composition and can be tailored to modify their mechanical properties. Hydrogels, which contain more than 30% water by weight, maintain structural integrity through physical and chemical crosslinks between polymer chains (Figure 10). They can be either biologically derived (e.g., alginate, chitosan, collagen, hyaluronic acid) or synthetic (e.g., polyethylene oxide, polyvinyl alcohol, polyacrylic acid, polypropylene fumarate-co-ethylene glycol). Matrigel, derived from mouse sarcoma ECM proteins, is the most commonly used naturally occurring hydrogel in cell biology (Ferreira et al., 2020). A key advantage of hydrogels is their ability to modulate stiffness and other physical properties, allowing the recreation of cell-ECM interactions (Liu et al., 2018), and providing a more accurate model for studying invasion and metastasis. Hydrogel-based models are also widely used in preclinical drug screening (Rijal and Li, 2017; Xu et al., 2022; Dang et al., 2022; Jie et al., 2022; Below et al., 2022), (Figures 8, 10).

### 11.3 *In vitro* tumor models based on patient-personalized dECMs

dECM-based models are excellent tools for drug screenings and personalized therapies (Ferreira et al., 2020). However, the heterogeneity of the dECM makes necessary the establishment of standardized protocols for tissue selection and decellularization. Given the ECM’s role in cancer therapy responses, particularly in radiotherapy, patient-derived dECMs have been instrumental in understanding how radiotherapy-induced ECM changes affect breast cancer cell behavior. These data demonstrate how radiotherapy can create an immunosuppressive microenvironment that can promote invasiveness by altering ECM characteristics (Zhu et al., 2024). These findings support the broader use of human and patient-derived dECMs in cancer models, offering more accurate preclinical evaluations and aiding in the development of personalized treatments (Figures 8, 10). Moreover, dECMs derived from human organs, such as the kidney, are successfully applied in regenerative medicine to promote tissue regeneration (Quinteira et al., 2024).

### 11.4 *In vitro* tumor models using dECM-based on tumor-on-a-chip

Tumor-on-a-chip models are devices that integrate microfluidics, tissue engineering, and microfabrication to replicate key features of tumor physiology. These models are particularly effective for studying dynamic cell-cell and time-dependent interactions (Miller et al., 2020). Incorporating dECM into tumor-on-a-chip systems provides platforms that better mimic the tumor microenvironment, allowing biomechanical and dynamic variations and enabling high-throughput drug screening applications (Figures 8, 10), (Ferreira et al., 2020). These models may potentially drive clinical decisions in patient follow-ups (Ooft et al., 2019). For instance, the combination of a patient-derived dECM with a microfluidic system allowed the development of *in vitro* drug screening models used to predict patient-specific tumor responses (Yi et al., 2019). Other studies have successfully integrated neuroblastoma cell lines and human endothelial cells within dECM on high throughput microfluidic chips, enabling controlled drug delivery and monitoring long term therapeutic effects (Liu et al., 2024). Similarly, Park and colleagues produced a vascularized lung tumor-on-a-chip model that integrated spheroids derived from a lung cancer cell line, umbilical vein endothelial cells and lung fibroblasts in hydrogels containing ECM elements (Park et al., 2021). Finally, a sarcoma model constructed on a 3D printed dECM connected to a microfluidic system demonstrated the role of ECM in regulating cell invasion (Dogan et al., 2024). These examples highlight how tumor-on-a-chip systems combined with dECMs, can significantly enhance drug testing accuracy (Figures 8, 10).

## 12 Discussion and future perspectives

Patient derived organoids have shown significant promise in cancer research due to their ability to closely mimic the physiology of the tumor from which they are derived. They can be efficiently developed from patient tissue samples, making them valuable for both translational applications and the development of personalized therapies (Drost and Clevers, 2018; Gilazieva et al., 2020). However, PDO models have several limitations, notably the absence of key elements of the TME, such as stromal cells, blood vessels and immune cells. This highlights the need for further studies focused on developing co-culture systems that incorporate these diverse cellular subpopulations to better reflect the *in vivo* environment (Yin et al., 2016). Single-cell RNA-sequencing analysis has significantly advanced our understanding of intra- and inter-tumor heterogeneity, revealing novel cell-cell and cell-ECM interaction pathways critical to tumor progression (Newman et al., 2019). Incorporating these insights into the development of 3D *in vitro* models is essential for improving the accuracy and resolution of tumor representations (Ferreira et al., 2020). Moreover, it is noteworthy that PDOs derived from advanced tumors tend to grow much slower than those from normal epithelium or early-stage tumors, probably due to their high rate of mitotic failure, which leads to cell death (Drost and Clevers, 2018).

Another important consideration for improving PDO models is the role of the ECM. The ECM significantly influences cancer progression by providing biochemical and biomechanical signals that regulate cell behavior, which includes proliferation, invasion, and resistance to therapy (Cox, 2021). However, traditional PDO culture methods often rely on xenogeneic ECM sources, like Matrigel, which fail to replicate the native tumor ECM composition and mechanical properties. Additionally, PDOs growth often requires the use of fetal calf serum for the production of WNT-conditioned medium. These two elements introduce extrinsic factors not present in the original tumor, potentially skewing results. This has prompted growing interest in human-derived and synthetic ECMs to more accurately mimic the tumor microenvironment (Gjorevski et al., 2016). Nonetheless, synthetic ECMs still require optimization and standardization. In particular, decellularization protocols need to be carefully standardized, as even slight variations in ECM composition can lead to significant changes in cellular phenotype. Single-cell analysis and mass spectrometry could play a pivotal role in refining dECM-based models (Newman et al., 2019).

The role of the ECM in tumorigenesis remains complex and not fully understood. The ECM composition varies widely across different solid tumor types, with differences in matrix deposition and stiffness contributing to this heterogeneity. Conflicting data regarding the role of ECM in cancer suggests that its influence on key tumor characteristics may not be applicable across all tumor types. Thus, further research is needed to uncover the dynamic biochemical and biophysical changes in the ECM during tumor progression, particularly those that lead to increased matrix stiffness, an essential factor in promoting various pro-tumor effects.

In conclusion, PDO models have proven their potential as valuable platforms for drug screening and for recapitulating the tumor microenvironment of individual patients. The integration of dECMs offers an exciting opportunity not only for the study of cancer cell-ECM interactions, but also for creating more personalized platforms for therapy testing.

## Footnotes

1
https://www.cancer.gov/tcga

2
https://hcmi-searchable-catalog.nci.nih.gov/

3
https://www.atcc.org/cell-products/collections-and-projects/human-cancer-models-initiative

4
https://pdmr.cancer.gov/
